# Removal or retention: evolving views on possible roles of the subacromial bursa in rotator cuff disease

**DOI:** 10.1530/EOR-2024-0183

**Published:** 2025-11-03

**Authors:** Yetian Ma, Wenwei Jiang, Pingkang Qian, Jiong Jiong Guo

**Affiliations:** ^1^ Department of Orthopedics and Sports Medicine, The First Affiliated Hospital of Soochow University, Suzhou, Jiangsu, PR China; ^2^ Department of Orthopaedic Surgery, Kunshan Hospital of Chinese Medicine, Kunshan, Jiangsu, PR China; ^3^ MOE China-Europe Sports Medicine Belt and Road Joint Laboratory, Soochow University, Suzhou, China; ^4^ MOE Key Laboratory of Geriatric Diseases and Immunology,Soochow University, Suzhou, China

**Keywords:** subacromial bursa, rotator cuff disease, pain, inflammation, mesenchymal stem cells, biological augmentation

## Abstract

- The subacromial bursa is located below the acromion, coracoacromial ligament, and deltoid deep fascia, above the rotator cuff and greater tuberosity of the humerus, and plays a crucial role in physiological processes such as exercise and pathological processes of rotator cuff diseases.
- The subacromial bursa is associated with inflammatory pain in patients with rotator cuff disease. Removing the bursa during surgery and intra-articular drug injection can both relieve this pain to some degree.
- Resection of the subacromial bursa improves intraoperative visualization and may loosen an already stiff shoulder joint; however, excessive resection appears to result in more severe adhesion.
- Current evidence suggests that the subacromial bursa is a source of reparative cytokines and mesenchymal stem cells that may contribute to and enhance the healing of the injured rotator cuff and improve prognosis.
- Research studies related to the utilization of the subacromial bursa for bioaugmentation is ongoing and shows potential to promote patient recovery.
- Preservation or bioaugmentation with the subacromial bursa during rotator cuff surgery might lead to a better prognosis, but there is not yet sufficient evidence to prove this.

## Introduction

The saccate synovial structure subacromial bursa (SAB), also known as the subdeltoid bursa, is located below the acromion, coracoacromial ligament, and deltoid deep fascia, above the rotator cuff (RC) and greater tuberosity of the humerus. As it is widely accepted that the SAB is crucial for the movement of the shoulder joint, it is often referred to as ‘a secondary shoulder joint’. RC tear (RCT), as a typical RC disease (RCD), is characterized by pain and limitation of movement. Conservative treatment may only relieve symptoms, while RC repair surgery is often considered the final choice for treating RCT. Under pathological conditions such as RCD that result in corresponding histopathological changes characterized by inflammatory hyperplasia and hypertrophy in the SAB, it may be responsible for pain and movement disorders (1). Thus, in common practice, surgeons typically remove the SAB partially or entirely to reduce postoperative pain for patients and obtain a better surgical field of view as well as pain management (2). However, other studies have claimed that the SAB exhibits more biological and histological repair effects than damage in promoting the healing of shoulder tendons (3, 4). This reparative effect of the SAB may be attributed to its secretion of reparative factors and its unique stem cell properties. Mesenchymal stem cells (MSCs) derived from it have been shown to have proliferation and differentiation capacity similar to bone marrow concentrate extracts (5). These research studies have shed light on a new method of biological augmentation using MSCs or other growth factors to treat RCD and accelerate patient healing (6). Despite the debate on the necessity of routine bursectomy in RC surgery, with new insights into the SAB, it seems that extensive debridement and bursectomy should give way to the preservation of the SAB at its anatomical position. This paper highlights the controversies and corresponding reasons for different managements with SAB during surgeries, reviews the local biological characteristics of the SAB in the pathogenesis and recovery of RCD, and showcases new progress and future targets in utilizing bursa tissue to promote RC tendon recovery. We find that preserving the SAB during RC surgery may lead to better RC healing when secondary pain is manageable. The clinical value of preserving the SAB or utilizing the SAB for bioaugmentation warrants further investigation.

## Materials and methods

This is a narrative review. We conducted the review search encompassing PubMed, MEDLINE, and Web of Science to identify pertinent articles, with the specific keywords including ‘subacromial bursa’, ‘rotator cuff’, ‘rotator cuff tear’, ‘rotator cuff repair’, ‘mesenchymal stem cell’, and ‘biological augmentation’. We review the advantages and disadvantages of intraoperative resection of the SAB in patients with RCD, and biological augmentation with the SAB is also included.

## Characteristics of RCD patients’ SAB in clinical practice

### Causes of pain and inflammation

**Figure 1:**
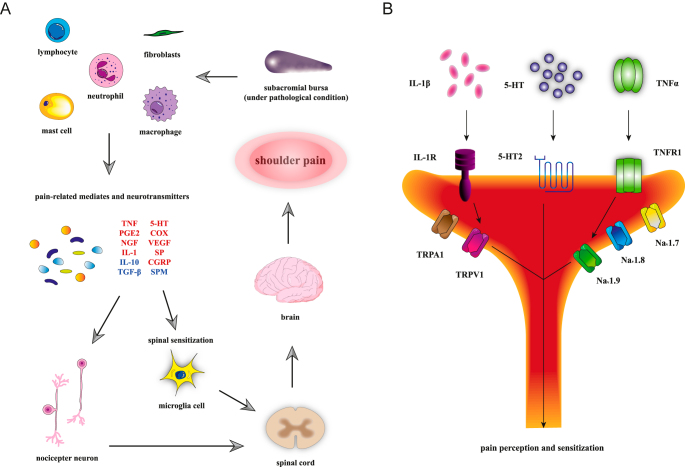
The SAB under pathological inflammatory conditions is a potential source of pain. (A) The role of the SAB in shoulder pain. Immune cells and other effector cells, such as fibroblasts, produce a series of pro-inflammatory and pain-related mediators (red), which are far more than the anti-inflammatory factors (blue) they secrete, thereby stimulating nociceptors to produce relevant signals. Some of these mediators are able to trigger central sensitization via microglia. All these signals are transmitted upward to the spinal cord and converge into the pain sensation in the brain, ultimately leading to local shoulder pain. (B) The signaling pathways involved in pain transmission and sensitization by specific cytokines and ion channels. Some inflammation factors and neurotransmitters participate in pain transmission pathways, while some of them can sensitize pain through ligand-gated channels (TRPV1, TRPA1) and voltage-gated sodium channels (Nav1.7, Nav1.8, Nav1.9) in the peripheral terminal of nociceptors.

**Figure 2:**
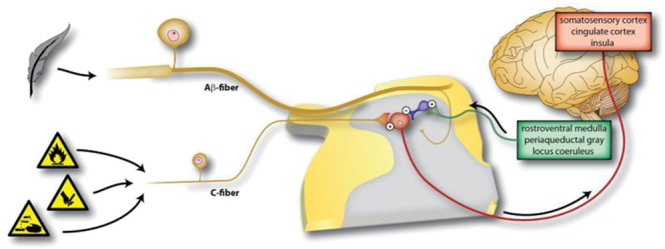
Central sensitization. Changes in the spinal cord that lead to the strengthening of synaptic input from both nociceptors and low-threshold mechanoreceptors onto nociceptive neurons contribute to an amplification of pain, with a reduction in its threshold, an expansion in its spatial extent, and a change in its temporal characteristics. The recruitment of normally innocuous afferent inputs to the nociceptive pathway is an important aspect of central sensitization. (Reprinted from von Hehn (19), 2012).

It is widely accepted that the SAB plays a crucial role in shoulder pain in RCD. Athletes with thicker SAB and those with neovascularization tend to experience much more pain (7). Hypertrophy, inflammation, edema, and necrosis of the SAB are all directly related to pain regardless of the presence of necrosis of the RC tendon (8). Under pathological conditions, the SAB activates immune cells and other effector cells to produce a series of inflammatory mediators and cytokines, thus stimulating nociceptors to generate relative pain signals (Fig. 1A). At the anatomical level, diffused and abundant free nerve tissue endings have been found in the SAB, especially around the anterior horn of the acromion and the greater tuberosity of the humeral head (9). This agrees with later findings that the density of nociceptors in the SAB is higher than in nearby tissue (10). The suprascapular and lateral pectoral nerves, as well as the axillary nerve, are involved in the innervation of SAB (11). As the most frequently identified innervation pattern, articular branches from these three nerve bridges would contribute to pain perception by triggering pain generation and signal aggregation. Apart from nerve conduction, the inflammatory changes of the SAB under pathological conditions such as RCD were witnessed, with a predominance of CD-2 and CD-11b mononuclear cells in the SAB of patients with bursitis (12). In the pathological state of the shoulder, specific immune cells, cytokines, lipid mediators, growth factors, and neurotransmitters all contribute to the neuronal pathways of pain sensation (13). In an experiment on IL-1-induced subacromial synovitis, the expression of cytokines in patients’ synovial specimens was significantly correlated with the degree of pain (14). Blaine *et al.* (15) saw an increasing expression of TNF, IL-1α, IL-1β, IL-6, MMP-1, MMP-9, COX-1 and COX-2 in the SAB in the RCT patient group, primarily in bursa-like cells. Of all the inflammatory factors, IL-1β, TNF-α, substance P (SP), and vascular endothelial growth factor (VEGF) are key in causing subacromial bursitis and inducing RCD-related pain. IL-1β is known to mediate NGF and COX-2 activity in human synovial fibroblasts and macrophages, thus modulating pain (16). Transforming growth factor-β (TGF-β)-activated kinase 1 (TAK1) suppression would reverse this process, indicating the crucial role of TAK1 in regulating pain (17). Besides the infiltration of inflammatory factors such as IL-1β and TNF-α, neuropeptide SP, which is widely distributed in nerve fibers, is also involved in the transmission and modulation of pain. It is mentioned that SP has been proved to be associated with pain in RC tendinopathy (18). VEGF, found to be elevated in the SAB in RCD, is believed to be associated with motor pain. After being stimulated and regulated by inflammatory mediators and neurotransmitters, nociceptive ion channels such as ligand-gated channels and voltage-gated sodium channels increase the release of action potentials, thereby sensitizing pain sensation (19). Microglia cells in the spinal cord activate cascade signaling through the production of mediators and cytokines, thus participating in pain generation and sensitization (13). A brief diagram of the cascade signaling pathways involved in pain transmission and sensitization by specific cytokines and ion channels is shown (Fig. 1B). In addition to peripheral sensitization, central sensitization can lower the threshold, ultimately leading to increased pain (Fig. 2) (19). These findings indicate that the SAB functions as a potential cause of pain in RCD through nerve conduction and inflammatory mediators, thus proving the feasibility of total or partial SAB resection.

### Influences of fibrosis and adhesion

The SAB seems to be related to fibrosis and adhesion of shoulder joints in RCD patients. Patients with adhesive capsulitis of the shoulder may experience aseptic exudation in the subacromial space owing to the SAB, ultimately resulting in joint stiffness and limited mobility (20). The SAB in patients with RCT with stiffness shows severe fibrosis and inflammatory hypertrophy, suggesting a relationship between the SAB and adhesion. Generally, factors contributing to fibrosis of the shoulder joint capsule and tendon, which ultimately lead to ankylosis and joint stiffness, can be concluded to corresponding upregulated growth factors caused by RCT and inflammatory reactions (21), substantially associated with the SAB. Microscopically, the inflammatory hyperplastic SAB generates a series of profibrotic signals through cellular interactions, leading to excessive extracellular matrix (ECM) production and ultimately to adhesion and joint stiffness. Fibrotic SAB after RC injury is partly reversed by microRNA-29a targeting COL3A1 mRNA (22), suggesting a possible mechanism. Yet there is insufficient evidence at the level of molecular mechanisms for the potential relationship between the SAB and fibrotic adhesions. A most intuitive and stable fibrosis pathway is that macrophages express fibrosis-associated factors that activate fibroblast transformation into myofibroblasts and engage them as effector cells to unfettered ECM deposition and sclerosis. Star cytokines capable of causing fibrosis similarly play a key role in fibroblast signaling in the SAB. The insistence on retaining the SAB, which produces adhesion and stiffness in the shoulder joint, in patients with RCD during surgeries seems to be a counterintuitive move. However, not all evidence supports the benefits of complete SAB resection for the release of shoulder adhesion after RC injuries, especially when no significant adhesions are found preoperatively. A randomized controlled trial was conducted to evaluate the effects of varying degrees of bursa resection during arthroscopic RC repair (23). In the extensive bursectomy group, the bursa was cleared to the anterior and posterior gutter areas and to the far medial part of the RC tendon and muscle. In contrast, the bursa in the limited bursectomy group was saved as much as possible. The final outcome indicated that the extensive bursectomy group could lead to more postoperative bursa thickening at 6 months and external rotation restriction at 1 year, along with the hiatus of a significant difference in pain reduction. The authors attributed this to the proliferation of the SAB and adhesion in the subacromial space caused by surgery. This study may suggest that excessive intraoperative debridement of the SAB may lead to more postoperative adhesion, thereby affecting the recovery of shoulder joint motion functions in patients. Therefore, from the perspective of RC injuries leading to adhesion and stiffness, and taking into account the available evidence, the practice of removing the SAB is not foolproof. Most surgeons believe that the more invasive procedures, such as excessive bursectomy, are performed in the subacromial space during RC repair, the more likely it is to develop scars or adhesions, resulting in early postoperative and even late stiffness (24). So, in patients with existing and persistent shoulder stiffness and limited motion, SAB debridement is essential for immediate symptomatic relief. However, when dealing with RCD patients without significant adhesion, one question must be answered: is excessive subacromial bursectomy appropriate, or would it increase the degree of adhesion in reverse? In the case of mild SAB lesions, clearing only the necessary part and avoiding excessive resection seems to be a better way to avoid postoperative fibrosis and shoulder adhesion.

### Barriers for visualization and convenience

Surgeons have routinely performed bursectomy for years to remove adhesion during subacromial surgeries to obtain a better surgical field of view and shorten the operative time (25). This consensus is reflected in the different types of RCD and the different surgical procedures chosen. In arthroscopic or open surgery for the treatment of subacromial impingement syndrome, besides subacromial decompression and RC repair, bursectomy is no doubt needed, considering better intraoperative visualization and inflammatory lesions clearance. Arthroscopic shoulder capsular reconstruction, as an alternative technique to solve severe RCT, necessarily includes the procedure of bursectomy to gain visual space for surgical operations. In various RCDs similar to the ones described above, the diseased SAB is often accompanied. Sarkar *et al.* (26) concluded that the SAB experienced proliferation or degeneration secondary to RCD due to the findings of thickened bursa, lipid globules, fibrous lamina along the inner nuclear membrane, and an increased number of vascular channel cells, without leukocytic infiltration of the saccular wall. The intraoperative field of view, whether arthroscopic or open, is greatly affected by the pathological changes of the SAB, such as inflammation, edema, hyperplasia, and calcification. Given that bursectomy with or without acromioplasty obtains similar therapeutic results in subacromial impingement (27) and RC calcific tendinopathy (28), the further pursuit of faster operating speed, shorter operating time, more precise surgical positioning, and better control of successful operation coincidentally points to the clearing of the SAB. This would be more convenient for optimizing the surgeon’s experience and, meanwhile, reducing patients’ anesthesia-related risks and surgical complications. In general, whether partial or total, necessary SAB resection is a fast and effective way to obtain visualization while balancing therapeutic effects in arthroscopic or open surgery.

### Sources of reparative cytokines

The SAB is additionally able to produce reparative cytokines, especially growth factors, involved in the treatment of RCD. The supernatant of the SAB may affect the repair process of tendons through growth factors, which are influenced by mechanical stress (29). A systematic review concluded the histological changes in RCD and identified molecular biomarkers found to be altered, including ECM enzymes, cytokines, growth factors, and neuronal signals (30). The authors reported an increase in growth factors such as TGF-β, bone morphogenetic protein (BMP)-2,7, basic fibroblast growth factor (bFGF, FGF-2), and VEGF.

Basically, BMP and TGF-β are involved in inflammatory processes and cell proliferation. Therefore, elevated expression of BMP and TGF-β in the SAB suggests their association with damage repair after RCD. BMP plays its biological role by inducing osteoblast and chondroblast differentiation. In RCT patients, it promotes regeneration and repair of the tendon-bone interface. BMP-2 combines with BMP receptor II and thus activates the downstream pathway of Smad1/5/8. Runx2 could help Smad proteins bind to the osteoblast-specific cis-acting element 2 (OSE2), thereby jointly regulating the expression of osteocalcin and osteoblast collagen. In addition, BMP-2 would regulate gene expression through the p38-MAPK pathway, leading to the synthesis of type II collagen regulated by SOX9 (31). Exogenous supplementation with BMP-7 and rhBMP-12 has demonstrated a reparative role in RCTs (32, 33). TGF-β promotes the synthesis of collagen, elastin, and fibronectin by fibroblasts, which are involved in ECM synthesis, inflammation, and injury repair. Similarly, TGF-β regulates osteogenic gene transcription through the Smad and p38-MAPK pathways, which both converge at the Runx2, and then promote the proliferation and differentiation of osteoprogenitor cells (34). Although functionally BMP and TGF-β contribute to cell proliferation, there appears to be evidence that excessive elevation leads to RCD progression rather than damage repair. A previous study suggested the presence of active BMP-2/4 and BMP-7 in the SAB, inducing ectopic bone and cartilage transformation and thus participating in the progression of RCD (35). However, Minkwitz *et al.* (36) confirmed BMPs in the bursa but did not see a difference in expression levels between the healthy and tear groups, which denied the negative impact of BMPs on prognosis.

In addition, one of the reasons why the SAB is rich in angiogenesis is its high expression of VEGF. The role of VEGF in angiogenesis has been abundantly studied and is unquestionable, and VEGF promotes tendon healing primarily by affecting angiogenesis (37). VEGF-A performs the dominant role among VEGF family in the regulation of angiogenesis, while VEGF receptor (VEGFR)-2 functions as the major receptor whose activation promotes angiogenesis and vascular permeability among all the VEGFRs (38). The binding of VEGF to VEGFR-2 leads to the latter’s dimerization, followed by activation of PLCγ within the cell, which then initiates the Ras – Raf – MEK – ERK signaling pathway, the classic MAPK pathway (39). Meanwhile, activation of the cascade reaction of the p38 nonclassic MAPK, as well as the phosphatidylinositol 3 – kinase (PI3K) – Akt pathway, together with classic MAPK transduction, ultimately leads to the survival, proliferation, and migration of vascular endothelial cells. Wang *et al.* (40) demonstrated the crucial effect of YAP/TAZ through the Hippo signaling pathway in VEGF angiogenesis. Mechanistically, VEGF activates YAP/TAZ through their dephosphorylation via modulation of SRC family kinases (SFKs), Rho GTPase activity, actin cytoskeleton dynamics, and large tumor suppressor gene 1 (LATS1) activity, thereby inducing angiogenesis. Interestingly, the BMP family has been reported to act as a regulator of the VEGFR2 signaling pathway, especially BMP-2 and BMP-6 (41). It is worth mentioning that BMP-2 induces a synergistic effect with VEGF, while BMP-6 leads to VEGF-mediated angiogenesis through the TAZ-Hippo signaling pathway. As one of the reparative cytokines in the SAB, VEGF actively participates in tendon healing after RCD.

FGF is related to bone and cartilage development as well as collagen synthesis. FGF-2 plays an important role in tendon-to-bone healing, cartilage repair, bone repair, and nerve regeneration (42). It promotes blood vessel formation and stimulates fibroblast proliferation, thereby inducing fibrinolytic zymogen and collagen synthesis, which are involved in tendon repair. FGF-2 can promote the synthesis of scleraxis (SCX) and tenomodulin (Tnmd), stimulating tendon-bone healing and improving biomechanics in RCT (43). A fibrin clot containing bFGF enhances the maturation and bioactivity of the tendon-bone interface, thus contributing to healing (44). Evidence of the capacity of FGF to synthesize ECM and collagen fibers suggests its role in the progression of tendon repair and tissue regeneration in RCD.

**Figure 3:**
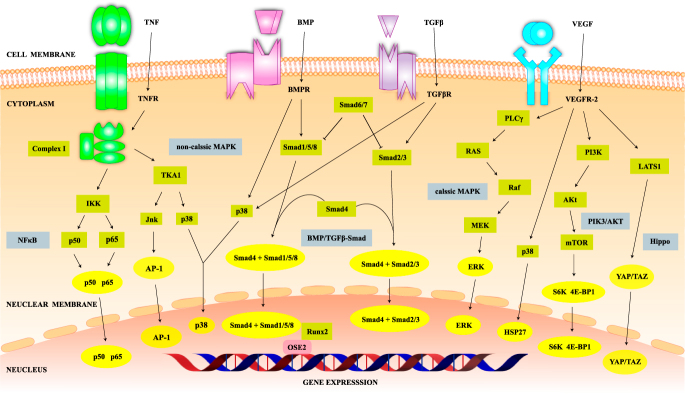
Signaling pathways of some cytokines and inflammation factors in subacromial bursitis.

The aforementioned BMP, TGF-β, VEGF, FGF, and other factors not mentioned but present in the SAB are categorized as facilitatory chemical mediators associated with the SAB. Characteristic features and crucial signaling pathways of these facilitatory chemical mediators, along with inflammation- and pain-related chemical mediators, and some inhibitory chemical mediators, are concluded in Table 1 and Fig. 3. The changes in matrix components caused by other inflammatory shoulder injuries or synovial bursa-related diseases have been summarized (Table 2) (4, 9, 12, 14, 15, 16, 17, 35, 36, 45, 46, 47, 48, 49, 50, 51). Potential tendencies toward vascular proliferation and reconstruction, inflammation regulation, increased ECM synthesis, and reparative potential may indicate the initial and developmental stages of regeneration in tendons and nutritional effects on the local microenvironment, providing a basis for the preservation of the SAB.

**Table 1:** Most critical chemical mediators involved with SAB.

Chemical mediators	Major characteristic features
Inflammation and pain related chemical mediators	
Pro-inflammatory cytokines	
IL-1, TNF-α	Initiates local inflammatory response and mobilizes immune cells; induces tissue inflammatory injury and repair; mediates inflammatory and neuropathic pain
Anti-inflammatory cytokines	
IL-6	Possesses both pro-inflammatory and anti-inflammatory effects; mediates sensitization of peripheral injury receptors involved in pain signal generation and transmission
IL-10	An immunosuppressive cytokine capable of inhibiting pro-inflammatory cytokine production; stimulates secretion of opioid peptides from microglia, thereby inhibiting pain
SPM	Reduce inflammation to restore tissue homeostasis
Neuropeptides	
SP	Acts directly on pain receptors, lowering their thresholds and making pain signals more easily activated
CGRP	Causes vasodilation and increases vascular permeability, which exacerbates pain
Prostaglandins	
PGE-2	Increases local blood flow and vascular permeability; stimulates nerve endings to increase sensitivity to pain
Cyclooxygenase	
COX-1, COX-2	Produced prostaglandins play an important role in pain perception
Others	
NGF	Promotes the growth and differentiation of nociceptors while increasing the sensitivity of nociceptors
VEGF	Involved in neuropathic pain through central sensitization; associated with inflammatory pain
Inhibitory chemical mediators	
Matrix metalloproteinase	
MMP-1, MMP-9	Activated in response to inflammation, capable of degrading ECM components such as collagen and fibronectin, leading to localized tissue degeneration and damage
Reactive oxygen species	
ROS	Causing oxidative stress, damaging cells, and tissues
Facilitatory chemical mediators	
Growth factors	
BMP-2, BMP-7	Promotes regeneration and repair of the tendon-bone interface; improves healing by modulating the inflammatory microenvironment
TGF-β	Promotes the synthesis of collagen, elastin and fibronectin by fibroblasts, which are involved in ECM synthesis and inflammation and injury repair; may cause tissue fibrosis and stiffness when overexpressed
PDGF	Promotes synthesis and remodeling of ECM; stimulates proliferation and migration of fibroblasts and tenocytes.; induces neovascularization
VEGF	Induction of vascular endothelial cell proliferation, migration and lumen formation; maintains vascular stability; possesses neuroprotective and restorative effects
IGF-1	Stimulates ECM and collagen synthesis, promotes fibroblast proliferation and migration
bFGF	Promotes blood vessel formation and stimulates fibroblast proliferation, thereby inducing fibrinolytic zymogen and collagen synthesis, which are involved in tendon repair

SPM, specialized pro-resolving mediator; SP, substance P; CGRP, calcitonin gene-related peptide; PGE-2, prostaglandin-2; COX, cyclooxygenase; NGF, nerve growth factor; VEGF, vascular endothelial growth factor; MMP, matrix metalloproteinase; ECM, extracellular matrix; ROS, reactive oxygen species; BMP, bone morphogenetic protein; TGF-β, transforming growth factor β; PDGF, platelet-derived growth factor; IGF-1, insulin-like growth factor-1; bFGF, basic fibroblast growth factor.

**Table 2:** Changes to ECM components in RCD or other bursa-related diseases (enzymes, cytokines, growth factors, neuronal factors, genes, and others).

Reference	Major findings in ECM components		Model	Specimen		
	Increased/positive	Decreased/negative		Type	EXP	CON
Santavirta *et al.* (12)	CD-2 and CD-11b mononuclear cells	PCA-1	Human, SAIS	SAB	12	-
Ide *et al.* (9)	SP, CCRP, RGP9.5	-	Human, shoulder dislocation or fracture	SAB	10	3
Gotoh *et al.* (45)	SP	-	Human, SB/RCT	SAB	37	7
Gotoh *et al.* (14)	IL‐1β, sIL‐1ra, icIL‐1ra	-	Human, SB/RCT	SAB	39	10
Yanagisawa *et al.* (4)	VEGF	-	Human, SB/RCT	SAB	50	-
Blaine *et al.* (15)	TNF, IL-1α, IL-1β, IL-6, MMP-1, MMP-9, COX-1, COX-2, SCYA24, SCYA2, SCYB10, SCYB5, SCYE1	-	Human, SB	SAB	14	4
Kim *et al.* (46)	SDF-1/CXCL12	-	Human, RCD	SAB	18	4
Molloy *et al.* (47)	mGluR5 and 6, NMDARL1, FLIP, HSP27, HST70, FGF receptor-1, IL-11, attractin	GRIP-1 and -2, PARP, FGF receptor-3, IL-2	Rat, TD	Tendon	24	12
Neuwirth *et al.* (35)	Collagen type I and III, BMP-2, BMP-7, FGF-2, VEGF, IL-1β, TNF-α, TGF-β	Aggrecan, collagen type II and X	Human, RCT	SAB	29	2
Millar *et al.* (48)	IL-18, IL-15, IL-6, MIF, TNF-α, caspases 3 and 8	-	Human, RCT	Tendon	17	10
	IL-6, IL-11, IL-15, IL-18, anti-NGF30, MIF, GATA-3, PAF-AH, CD3 γ chain, IgG-2b gene, T-cell receptor variable β chain, attractin, TNF-α receptor, RANKL, HSP-27, cFLIP receptor, caspase 8	IL-2, Ig heavy chain, CD94, T cell receptor α chain, T-cell receptor, HIF-1, type-2 angiotensin II receptor, PARP	Rat, TD	Tendon	12	12
Franklin *et al.* (49)	Glutamate, NMDAR1, mGluR2	mGluR7, NK-1, BDKRB2, TH	Human, RCT	Tendon	64	16
Takano *et al.* (16)	IL-1β, TNF-α, NGF	-	Human, OA	SYN	25	-
Nagura *et al.* (17)	COX-2, NGF	-	Human, RCT	SAB	18	-
Farfaras *et al.* (50)	CD-72, TNF-α	CD-3, IL-6	Human, SAIS	Capsule	8	12
	IL-6, TNF-α	CD-3, CD-72		Tendon		
Minkwitz *et al.* (36)	IL1β, TNF-α, COX2, MMP-1	IL-10, BMP-2, MMP-9, MIF, PENK, GAP43, PGP9.5	Human, RCT/OD	SAB	29	5
Miura *et al.* (51)	COL3, scleraxis, MMP-13, IL-1β, iNOS, IL-10, Arg-1	-	Rat, RCT	SAB	21	-

RCT, rotator cuff tear; SB, subacromial bursitis; OA, osteoarthritis; OD, osteochondral degeneration; TD, tendon degeneration; SYN, synovial tissue; SDF-1, stromal cell-derived factor-1; GATA-3, globin transcription factor binding protein 3; PAF-AH, platelet-activating factor acetylhydrolase; RANKL, receptor activator of NF-kb ligand; SCY, small inducible cytokines; PGP9.5, protein gene product 9.5; HSP-27, heat shock protein-27; cFLIP, cellular FLICE inhibitory protein; HIF-1, hypoxia-inducible factor 1; mGluR, metabotropic glutamate receptor; NMDAR, N-methyl-D-aspartate receptor; NMDARL1, NMDA receptor-like 1; GRIP, glutamate receptor interacting proteins; PARP, poly(ADP-ribose) polymerase; HST, testis heat shock-related protein; NK-1, neurokinin-1; BDKRB2, bradykinin receptor B2; TH, tyrosine hydroxylase; iNOS, inducible nitric oxide synthase; Arg-1, arginase-1; Exp, experimental; CON, control.

### Potentials of MSC and progenitor cell properties

**Figure 4:**
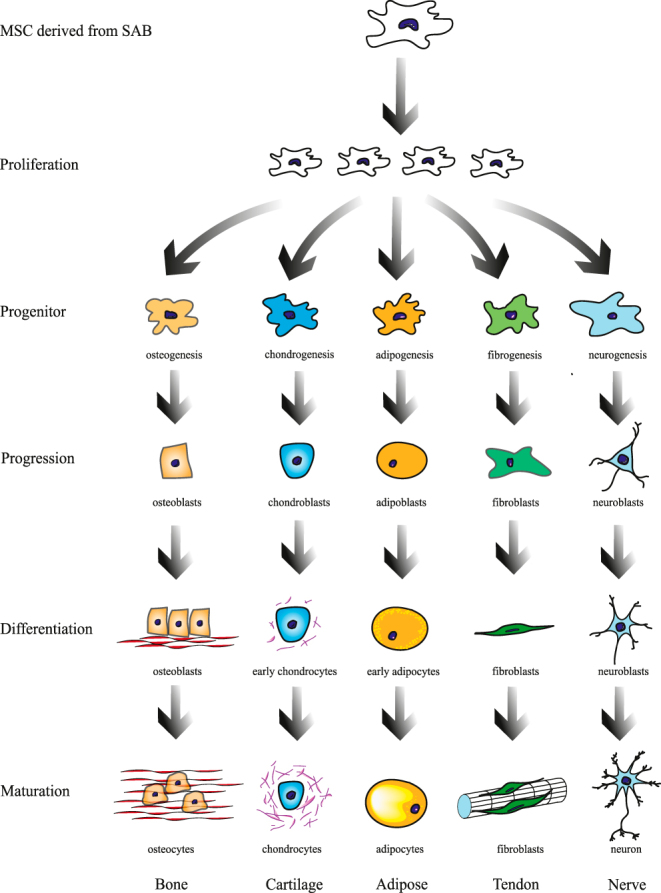
Differentiation potential of MSCs derived from SAB. MSCs isolated from SAB have the ability to proliferate and differentiate into bone, cartilage, adipose, tendon, and nerve under different culture and induction conditions.

MSCs have broad prospects in hematopoiesis, immunology, and regenerative medicine due to their confirmed potential for multidirectional differentiation and self-replication. In studies of RCI treatment, MSCs are usually derived from bone marrow, adipose tissue, tendon, and umbilical cord blood. MSCs have achieved preliminary success in tendon regeneration, pain downregulation and shoulder function improvement by intratendinous injection (52, 53). The presence of MSCs in originally discarded SAB was first discovered in 2013 and was also characterized in SAB-derived cells of mice (54). Bursa-derived MSCs have superior viability, differentiative capacity, proliferation, and colony-forming abilities compared to cells derived from the supraspinatus tendon and enthesis (55). These SAB-derived cells also show good outcomes in chondrogenesis, osteogenesis, and adipogenesis research studies (55). Later, in another research by Song *et al.* (56), bursa-derived MSCs treated with BMP-12 turned out to express markers of tenocytes and form tendon-like tissue with parallel collagen fibers when seeded onto scaffolds *in vivo*, indicating the differentiation potential of tendons. As a result, SAB seems to be a promising source of MSCs to promote tendon healing in the treatment of RCT. After the induction of neurogenic differentiation, as reported by Aydın *et al.* (57), positive neuron/glial cell-specific markers were detected. The characteristics of neural differentiation allowed SAB to be a promising MSC source for neurodegenerative diseases. The differentiation ability of MSCs extracted from the tissue of the SAB can be summarized as follows (Fig. 4). Large numbers of progenitor cells were found in the SAB and surrounding matrix of patients undergoing RC repair surgery, especially over the tendon and muscle belly of RC (58). This sheds light on biological treatment taking advantage of the pluripotent stemness of SAB-derived MSCs and supports the preservation or reuse of SAB during RC surgeries. Morikawa *et al.* (59) presented the viewpoint that the source of MSCs affected their capabilities, for cells derived from bone marrow aspirate exhibited inferior proliferation and differentiation characteristics compared to those from SAB. Furthermore, even SAB located near different parts of shoulder tissues may exhibit different biological characteristics. It was found that the SAB originally attached to the RC tendon rather than the muscle generated more colonies, indicating greater proliferation ability of RC tendon (59). Distinct from the fatty infiltration of SAB in the muscle zone, SAB in the tendon zone was predominantly fibrotic hyperplasia. SAB cells had a greater capacity to proliferate in the tendon zone than in the muscle zone, and this feature of anatomical location influencing proliferation potential may originate from the peri-tissue SAB-derived MSCs (60). In addition, some studies have mainly focused on factors that affect SAB-derived MSC activity. Muench *et al.* (61) reported in their research that the high proliferation potential of SAB cells was independent of patients’ demographic factors, RCT characteristics, and degree of shoulder joint degeneration. This finding was partly consistent with the later study by Morikawa *et al.* (62) in demographic characteristics. However, at the level of RCT, this study demonstrated a conspicuous negative relationship between the tear size and colony-forming potential. Morikawa *et al.* attributed it to higher specific cellular activity indicators. Another study showed that tendon-side SAB obtained from re-revised arthroscopic RC repair had significantly weaker proliferative capacity than those from initial RC repair (56). However, the osteogenic, chondrogenic, and adipogenic MSC properties were still preserved. Anyway, MSCs derived from SAB have certain potential to promote tissue healing in the subacromial space, regardless of patient characteristics or the number of surgeries performed. In summary, the MSC feature of SAB suggests the feasibility and advantages of retaining SAB.

## SAB: intra-articular drug injection

In patients with RCD, and especially in patients with RCT, damage to subacromial structures or repeated impingement produces aseptic inflammation, resulting in subacromial pain syndrome. In this case, the physiologic state of the SAB undergoes corresponding histopathologic changes, usually accompanied by subacromial bursitis, which releases a large number of chemical mediators involved in disease progression. Initial conservative treatment options for patients with pain and subacromial bursitis include rest and oral NSAIDs, while intra-articular drug injections may be an effective option for patients with uncontrolled pain. Steroid, hyaluronic acid, or platelet-rich plasma (PRP) injections into the SAB are among the common treatments used clinically to relieve pain and improve function in patients with RCT (63). Functionally, several studies have shown that intra-articular injections of hyaluronate (64, 65, 66), corticosteroids (67, 68, 69, 70), and PRP (71, 72) are effective in improving pain and shoulder function in patients with RCD. In terms of effects on the SAB, hyaluronate injections work by improving lubrication within the bursa, while corticosteroid injections provide pain relief through anti-inflammatory effects. PRP acts as an anti-inflammatory and pro-restorative agent with its high concentration of platelets and growth factors, further altering the pathology of SAB and stimulating healing of the tendon-bone interface in RCT. However, the vast majority of studies have focused on pain and shoulder function, with relatively little evidence of improvement in SAB in terms of morphology, pathobiology, and molecular biology. Hyaluronate accelerates tendon-bone healing after RCT and may be attributed in part to activating the chondrogenic capacity of MSCs (73). This is possible evidence that hyaluronate affects MSCs in SAB. Steroid intra-articular injections in patients with RCT were found to reduce SAB thickness after ultrasound evaluation and were more effective in patients with subacromial bursitis compared to the normal bursa group (74). Stromal cell-derived factor 1 (SDF-1, CXCL12), which is increased in SAB of patients with subacromial bursitis, is able to be downregulated by steroids (46). A recent study showed that the delivery of polymer microspheres containing dexamethasone to the intact bursa of rats improved the inflammatory state of the SAB with damaged tendons (75). In an *in vitro* model of RCT, treatment of the SAB with autologous active serum, PRP, or a combination of both resulted in an increase in anti-inflammatory M2 macrophage markers and a decrease in pro-inflammatory M1 macrophage markers (76). This evidence demonstrates that intra-articular drug injections can improve the pathology of SAB, reduce the inflammatory response, relieve pain, and promote tissue repair.

## SAB: total removing, partial or complete retention

SAB is considered to have great potential as a biological enhancer due to its high content of certain bioactive substances. The effectiveness of retaining the SAB alone for tendon repair has been verified. The positive interaction between SAB and tendon was revealed by *in vitro* co-culturing to mimic the *in vivo* inflammatory environment of RC (77). Dyrna *et al.* (78) demonstrated the superiority of human SAB in engraftment and survival compared with human bone marrow MSCs. It is noteworthy that the presence or absence of inflammation in the SAB did not significantly affect its ability to promote tendon-bone healing and biomechanical recovery (79). These give the clinical application of SAB retention. Thus, the current management exploration has been shifting to the feasibility of preservation of the SAB. Many animal experiments or clinical trials are attempting to identify the advantages and disadvantages of complete resection, partial retention, and complete retention of SAB for the recovery of patients with RCD. Sun *et al.* (80) found that preserving the SAB intact was more beneficial for RC regeneration in rats with supraspinatus tendon defects relative to excision of the SAB, as evidenced by improvements in both biomechanics and tendon histology. Another study also demonstrated a positive effect on RC healing by increasing vascularity and collagen with complete preservation of the SAB versus resection (81). Wang *et al.* (82) proved that rather than complete removing, anatomical and interpositional bursa preservation between tendons facilitated better recovery of tendon and bone. However, the degree of preservation, that is, partial or complete retention of the SAB, did not show significant differences in promoting tendon-bone healing. A randomized controlled study comparing extensive and limited synovectomy found that both yielded similar postoperative pain improvement and repair integrity. However, limited synovectomy resulted in better external rotation recovery and less bursal thickening at 6 months and 1 year postoperatively (23). The above evidence supports that either partial or complete retention of the SAB results in better clinical outcomes than complete resection of the SAB. However, fuller and more reliable evidence is still lacking before a final conclusion can be reached.

## SAB: intraoperative biological augmentation

Given the initial success of SAB retention in clinical trials, attempts have been made to more effectively treat and process the SAB in order to achieve biological reinforcement for tendon repair in RCD in clinical practice. Freislederer *et al.* (83) introduced a new arthroscopic sewing technique with PDS sutures in RC repair with the aid of SAB, which allowed the highly vascularized and mobilized parietal bursal layer to cover the tendon footprint. This bursa augmentation is free of specific technical risk; however, it may face difficulties in operating as well as final effects because of the fragile lateral aspect of the bursa. Bhatia *et al.* (84) also introduced an advanced SAB-to-tendon suture technique with preserved vasculature. Learning from previous lessons, they avoided damage to the flimsy lateral aspect of the bursa during collection and suturing, opting instead for the thicker, structurally stronger posterosuperior and lateral SAB. This application finally forms a tendon-bursa unit with a vascular bursal duvet and thus enables the fulfillment of tendon repair in RCD. Based on this modified technique, they later combined the SAB with biceps augmentation for massive cuff tears (85). Autografting of the relatively intact long biceps tendon provides structural strength by replacing the retracted and atrophied tendon in large RCT. At the same time, the grafted SAB exerts its regenerative potential, providing a superior healing environment and reducing the possibility of re-tearing. Furthermore, some people have started researching how to more efficiently utilize SAB to obtain cells with repair significance. In a study on isolating SAB-derived cells, the chopping group could obtain distinctly more cells than the whole SAB, and the detection of MSC markers was confirmed (86). Based on this, the new processing method of using SAB for biological augmentation is constantly developing. Pancholi *et al.* (87) optimized the surgical procedure and avoided suturing of the bursa. SAB was chopped by an oscillating shaver, collected afterward, and injected onto the surface of the shoulder RC. Another bio-enhancement technique called local autologous stem cell application (LASCA) for arthroscopic RC repair is described (88). During the surgery, the SAB was harvested, processed, and re-injected back into the vicinity of the tendon for biological enhancement. Both of the new intraoperative biological augmentations with SAB are based on developed processing methods and the advantages of MSCs, as well as reparative factors in the SAB tissue. Unfortunately, the only drawback is that there have been few further reports on the effectiveness of the above-mentioned new methods of SAB reimplantation and the prognosis of patients. This limits the clinical translational application of SAB biological augmentation. A retrospective cohort study found that the use of churned SAB reimplanted onto the surface of the lateral bursa of the tendon for bioenhancement did not have clinically relevant negative effects on patients (89). One preliminary study demonstrated high clinical effect at 1 year after operation in the improvement of function in patients with the aid of SAB through RC repair (90). This study used a so-called ‘Mega-Clot’ biologic scaffold combining autologous SAB, concentrated bone marrow aspirate (cBMA), and PRP. However, considering the restorative effect of cBMA and PRP themselves, the exact effectiveness of SAB remained to be identified. The researchers later improved the scheme and proposed a new method of arthroscopic biological augmentation with the combination of autologous SAB tissue, cBMA, PRP, platelet-poor plasma, and bovine thrombin (91). Although its clinical effect with SAB has not been further investigated.

## Discussion

The SAB is of significant importance in shoulder injury due to its distinct anatomical and biological attributes. Previous knowledge rests on SAB being a tissue that is lubricated in the physiological state, with inflammatory infiltration in the pathological state, and involved in adhesion and pain. The cellular landscape of SAB mainly includes fibroblasts, endothelial cells, mural cells, and immune cells. Its unique cell type composition and histological change may suggest its role in health or RCD. In current surgeries for RCD, it has been a common routine to perform SAB resection for inflammatory pain control and better surgical field of view acquisition. In fact, the overwhelming evidence described above has supported the conventional wisdom that SAB is indeed a significant source of inflammatory factors and is associated with fibrosis and shoulder stiffness. However, recent concepts suggest a potential therapeutic role for SAB in pathological states and the disadvantages of over-removing the SAB. Tissue-repair-friendly MSCs and secreted tissue repair factors have been discovered in SAB. SAB-derived MSCs are pluripotent and easy to obtain, and have great potential in RCD tendon-bone repair. SAB is able to secrete growth factors such as BMP, TGF-β, VEGF, and FGF, which play key roles in vascular remodeling, collagen synthesis, homeostasis maintenance, and tendon-bone healing. The proliferative and pluripotent nature of the SAB cells can help accelerate the repair of tendon tears in patients with RCD via various pathways such as chondrogenesis, osteogenesis, and tendon formation. Thus, excessive excision of the SAB reduces MSCs and the release of reparative factors, which is adverse to the repair of tendons and cartilage. Furthermore, in conjunction with the use of intra-articular injections of therapeutic substances such as hyaluronic acid, steroids, and PRP, it is possible to reduce SAB-related inflammatory pain as well as joint adhesions to some extent while retaining restorative properties of SAB. This further accelerates the clinical adoption of SAB. Although only a small number of animal experiments and clinical trials have examined the differences between fully preserved, partially preserved, and completely removed SAB in the prognosis of patients with RCD, the available evidence suggests that fully or partially preserved SAB results in better tendon-bone healing, pain improvement, and mobility compared with complete resection. This sheds light on clinical retention of SAB in RCD patients during shoulder surgeries.

Besides the activity of reparative factors and MSCs derived from SAB, SAB is preferred in clinical practice for its accessibility and economics. Therefore, it can be seen that the exploration of new techniques for preservation and biological augmentation with SAB is currently underway. Retaining the SAB or moving a structurally stronger portion of the SAB onto the tendon footprint utilizes the rich vascular structure of the SAB to create a suitable healing environment. In other cases, SAB is collected during surgeries, chopped or digested, or supplemented with drugs such as cBMA or PRP and re-injected into the paratendon for therapeutic effects of enriched stem cells and reparative factors. Despite the current emergence of many biological augment therapies inspired by the characteristics of the SAB, future treatments should pay more attention to the clinical efficacy and the characteristics of the bursa itself in RCD. Generally, more clinical results on these techniques remain to be presented to determine the *in vivo* behavior of the SAB and the effectiveness of biological augmentation. Anyway, it cannot be denied that increasing attention is focused on the repairing properties of SAB, and people are trying to explore the superiority of retaining SAB as a biological augmentation method in RCD treatment. The importance and feasibility of retaining SAB or utilizing SAB for biological augmentation are once again supported. The recent literature has again demonstrated histologically and biochemically that preservation of the SAB causes the tendon healing process after RCT to undergo a more appropriate inflammatory and reparative phase (51). Anyway, preservation and biological augmentation should be underlined in clinical practice, and it is recommended to avoid resection of lesion-free SAB to achieve better recovery and to control possible SAB-associated inflammatory pain and joint adhesions with the aid of intra-articular injections of drugs.

There are certain limitations of this review. Despite the advantages and disadvantages of SAB in RCD mentioned, other possible characteristics of SAB have not been discussed. The mechanoreceptive and proprioceptive abilities of the SAB may help to protect the shoulder joint from damage by promoting coordination, which may lack discussion. There are insufficient comparisons on the efficacy of SAB with complete retention, partial retention, or complete resection in patients with RCD. The reason for this may be that there is a paucity of the relevant research literature to support the corresponding conclusions as reliable and sufficient evidence. In addition, this study is not a systematic review, so lack of strictness and structure is unavoidable. Finally, bias generated during the literature search and uneven quality of the literature may affect the conclusion.

## Conclusion

This paper reviews the advantages and disadvantages of intraoperative resection of the SAB from the perspectives of inflammation, pain, fibrosis, visualization, reparative cytokines, and MSC properties. Intra-articular therapeutic drug injections can relieve the inflammation of the SAB in the RCD to a certain extent, reduce the inflammatory pain, and restore shoulder function. The results of limited animal experiments and clinical trials suggest that preservation of SAB correlates with a better prognosis. This may portend the potential of retaining SAB as a biological enhancement, but further evidence is needed to prove it. Finally, the progress of SAB biological augmentation during surgery is listed, and the feasibility of retaining the SAB is also included. The reparative cytokines it produces and its MSC potential make it a highly promising structure for promoting the tendon healing of RCD patients. Given the available evidence and information, we find that retaining the SAB seems to show some advantages in routine RCD surgery, despite the rationale for removing the SAB. Whether or not to consider retaining the SAB specifically in the clinic in anticipation of better healing is something that needs to be confirmed by more adequate studies and solid evidence.

## ICMJE Statement of Interest

The authors declare no conflicts of interest. The funders had no role in the design of the study; in the collection, analyses, or interpretation of data; in the writing of the manuscript; or in the decision to publish the results.

## Funding Statement

This work was supported by the  National Key Research and Development Program of China (grant number 2022YFE0199900), National Nature Science Foundation of China (grant number 62475181), Jiangsu Province Science and Technology Innovation Support Plan Project (grant number BZ2022051), China-Europe Sports Medicine Belt-and-Road Joint Laboratory, Ministry of Education of PRC (grant number 2023297), Project of MOE Key Laboratory of Geriatric Diseases and Immunology (No. KJS2607 ), and Key Research Project of Higher Education Teaching Reform of Soochow University (grant number 2023-12). The funders had no role in the design and conduct of the study; collection, management, analysis, and interpretation of the data; preparation, review, or approval of the manuscript; and decision to submit the manuscript for publication.

## Author contribution statement

YM and WJ performed the literature search, selected the articles, and extracted data. YM and JJG identified themes, summarized results, and wrote the manuscript. All authors approved the final version of the manuscript. JJG, on behalf of all authors, takes responsibility for the integrity of the work, from inception to manuscript.
